# An ancient and conserved function for Armadillo‐related proteins in the control of spore and seed germination by abscisic acid

**DOI:** 10.1111/nph.13938

**Published:** 2016-04-04

**Authors:** Laura A. Moody, Younousse Saidi, Daniel J. Gibbs, Anushree Choudhary, Daniel Holloway, Eleanor F. Vesty, Kiran Kaur Bansal, Susan J. Bradshaw, Juliet C. Coates

**Affiliations:** ^1^ School of BiosciencesUniversity of BirminghamBirminghamB15 2TTUK

**Keywords:** abscisic acid (ABA), *Arabidopsis thaliana*, Armadillo proteins, evolution, germination, moss, seed, spore

## Abstract

- Armadillo‐related proteins regulate development throughout eukaryotic kingdoms. In the flowering plant *Arabidopsis thaliana*, Armadillo‐related ARABIDILLO proteins promote multicellular root branching. ARABIDILLO homologues exist throughout land plants, including early‐diverging species lacking true roots, suggesting that early‐evolving ARABIDILLOs had additional biological roles.
- Here we investigated, using molecular genetics, the conservation and diversification of ARABIDILLO protein function in plants separated by *c*. 450 million years of evolution.
- We demonstrate that ARABIDILLO homologues in the moss *Physcomitrella patens* regulate a previously undiscovered inhibitory effect of abscisic acid (ABA) on spore germination. Furthermore, we show that *A. thaliana* ARABIDILLOs function similarly during seed germination. Early‐diverging ARABIDILLO homologues from both *P. patens* and the lycophyte *Selaginella moellendorffii* can substitute for ARABIDILLO function during *A. thaliana* root development and seed germination.
- We conclude that (1) ABA was co‐opted early in plant evolution to regulate functionally analogous processes in spore‐ and seed‐producing plants and (2) plant ARABIDILLO germination functions were co‐opted early into both gametophyte and sporophyte, with a specific rooting function evolving later in the land plant lineage.

## Introduction

Plant lifecycles undergo an alternation of generations between a haploid gametophyte and a diploid sporophyte phase (Hofmeister, 1851). In the earliest‐diverging land plants, the bryophytes, the gametophyte generation is dominant in the lifecycle, with the sporophyte being a relatively transient structure that remains attached to and largely dependent on the gametophyte (Glime, 2013). By contrast, in extant vascular plants, the sporophyte has assumed the dominant role, the most extreme example being in seed plants (spermatophytes), where the gametophyte is reduced to a few cells that are fully surrounded by sporophyte tissues (Evert & Eichhorn, 2012). Despite the different origins of the plant body in bryophytes and flowering plants, both lineages possess rooting and shooting structures, as well as dispersal structures (spores or seeds) that allow transition from one generation to the next and enable species propagation and distribution (Pires & Dolan, 2012).

The evolution of rooting systems was a key innovation enabling plants to be sessile, allowing absorption of nutrients and water, anchorage of the plant to its substrate, and responses to internal and external signals. Bryophytes possess simple hair‐like rooting structures called rhizoids (Jones & Dolan, 2012). Work in model bryophytes, the moss *Physcomitrella patens* (Prigge & Bezanilla, 2010) and liverwort *Marchantia polymorpha* (Shimamura, 2015), has demonstrated that rhizoid development has mechanistic similarity with the development of epidermal root hairs on the multicellular roots of the flowering plant *Arabidopsis thaliana* (Menand *et al*., 2007; Proust *et al*., 2016). This suggests that nonhomologous, but functionally similar, epidermal structures (i.e. tip‐growing cells with a rooting function) are regulated by genes that were co‐opted into both gametophyte and sporophyte (Menand *et al*., 2007; Proust *et al*., 2016). However, it is likely that a ‘rewiring’ of the root hair/rhizoid gene regulatory network occurred between the bryophyte gametophyte and the flowering plant sporophyte (Yi *et al*., 2010; Jang & Dolan, 2011; Jang *et al*., 2011; Pires *et al*., 2013; Tam *et al*., 2015).

Multicellular ‘true’ roots first evolved in vascular plants, independently in lycophytes and the fern/spermatophyte lineages (Banks, 2009; Jones & Dolan, 2012; Pires & Dolan, 2012). The most complex branched rooting systems, consisting of a primary root formed in the embryo, from which lateral roots (LRs) later arise, are present in ferns and in seed plants (Jones & Dolan, 2012; Pires & Dolan, 2012). Auxin plays a key role in promoting all stages of LR development (Peret *et al*., 2009; Lavenus *et al*., 2013), while many other plant hormones regulate various aspects of root branching (Osmont *et al*., 2007; Nibau *et al*., 2008; Fukaki & Tasaka, 2009). Moreover, ‘intrinsic’ regulators of LR development, which are not influenced by hormones, also exist (Malamy & Benfey, 1997; Nibau *et al*., 2008).

One example of an ‘intrinsic’ regulator of root branching in *A. thaliana* is the ARABIDILLO protein family, which shares structural similarity with the key animal Armadillo/β‐catenin developmental regulators (Coates, 2003). We have previously demonstrated a role for *A. thaliana* ARABIDILLO proteins in promoting LR formation (Coates *et al*., 2006). ARABIDILLO proteins are unstable, being turned over by the proteasome (Nibau *et al*., 2011), and their sequences are highly conserved across land plants and charophyte algae, including species that lack LRs, namely *P. patens*,* Selaginella moellendorffii* and *Klebsormidium flaccidum* (Nibau *et al*., 2011; Moody *et al*., 2012; Hori *et al*., 2014). By contrast, homologues of the ARABIDILLO‐interacting transcription factor *At*MYB93, which is part of a negative feedback loop that inhibits LR development, are not found outside flowering plants (Gibbs & Coates, 2014; Gibbs *et al*., 2014). These data suggest that ARABIDILLO proteins had additional, early‐evolving functions in plants.

Here, we addressed this possibility by examining the function of *ARABIDILLO* homologues (*PHYSCODILLO* genes) in *P. patens*. *Physcomitrella patens* has three *PHYSCODILLO* genes that probably have redundant functions (Moody *et al*., 2012). We define novel functions for PHYSCODILLO proteins in regulating spore germination in response to abscisic acid (ABA), an ancient hormone found across eukaryotes (Hanada *et al*., 2011; Takezawa *et al*., 2011). Furthermore, we show that *A. thaliana* ARABIDILLO proteins perform the analogous function in seeds. Our data suggest that ARABIDILLO homologues were co‐opted into both the sporophyte and gametophyte very early in land plant evolution to regulate germination processes via a network involving ABA, and that early‐diverging ARABIDILLO homologues already had the potential to regulate multicellular root development, a later‐evolving function of this protein family requiring interaction with flowering plant‐specific proteins.

## Materials and Methods

### Moss growth and culture

*Physcomitrella patens* (Hedw.) B.S.G. ssp *patens*, ecotype ‘Gransden 2004’ was obtained from Dr Andrew Cuming (University of Leeds, Leeds, UK). The *physcodillo2* deletion mutant has been described previously (Moody *et al*., 2012). Protonemata, gametophores and germinating spores were cultured as in Moody *et al*. (2012). The spore germination medium was additionally supplemented with 10 mM CaCl_2_ and 5 mM ammonium tartrate. The *physcodillo2* deletion mutant was maintained on BCD medium additionally supplemented with 20 μg ml^−l^ hygromycin B. The *physcodillo1a/1b/2* triple deletion mutant was maintained on BCD medium additionally supplemented with 20 μg ml^−l^ hygromycin B and 50 μg ml^−l^ G418.

### *Arabidopsis thaliana* growth and culture

For *in vitro* culture, *Arabidopsis thaliana* (L.) Heynh. seeds were sterilized in 20% Parozone bleach (Jeyes, Thetford, UK) for 15 min and then washed three times in sterile water. Seedlings were grown in long‐day conditions on 0.5× Murashige and Skoog (MS) medium, 1% agar, pH 5.7 ± 50 μg ml^−l^ kanamycin. Mature *A. thaliana* plants were grown in Levington M3 compost/vermiculite (Levington Horticulture, Ipswich, UK) in the glasshouse at 22°C under long days before harvesting of mature siliques.

### *Physcomitrella patens* transformation

Transformation was carried out as in Moody *et al*. (2012). Stable transformants were selected using 20 μg ml^−l^ hygromycin B and 50 μg ml^−l^ G418.

### Preparation of DNA and RNA

*Physcomitrella patens* genomic DNA was prepared as in Moody *et al*. (2012) and used directly in PCR reactions. Transforming plasmid DNA was prepared using the Qiagen Plasmid Midi Kit according to the manufacturer's instructions. *Physcomitrella patens*,* Selaginella moellendorffii* (hieron) and *A. thaliana* RNA was prepared using the RNeasy plant mini‐prep kit (Qiagen). RNA was treated with TURBO^™^ DNase (Life Technologies, Waltham, MA, USA) and then converted to cDNA using Superscript^™^ II reverse transcriptase and Oligo dT (Invitrogen).

### Generation and imaging of protoplast transformation constructs

*pUBI*::PHYSCODILLO‐GFP constructs were created by ligating the *PHYSCODILLO* cDNA sequences (Moody *et al*., 2012) into the multiple cloning site of the vector pUbi‐MCS‐GFP‐108‐II (GenBank KR297238). Generation of *pHSP*::PHYSCODILLO‐GFP constructs is outlined in Supporting Information Methods S1. *p35S*::ARABIDILLO‐GFP was used as in Coates *et al*. (2006). *p35S*::SELAGIDILLO‐GFP was derived from *SELAGIDILLO* cDNA (Moody *et al*., 2012) as in the ‘Construction of *arabidillo* mutant rescue lines’ subsection later. For transient expression, *pUBI*::PHYSCODILLO1‐GFP, *pUBI*::PHYSCODILLO2‐GFP, *p35S*::ARABIDILLO‐GFP and *p35S*::SELAGIDILLO‐GFP constructs were each transformed into wild‐type *P. patens* protoplasts multiple times. To verify nuclear localization, *pHSP*::PHYSCODILLO1‐GFP and *pHSP*::PHYSCODILLO2‐GFP were co‐transformed into *P. patens* protoplasts alongside *pHSP:*:NLS‐mCherry‐10835SNPT (GenBank KP893620). Following transformations, protoplasts were incubated overnight at 22°C before capturing images using a Leica SP2 inverted confocal microscope.

### Generation of PHYSCODILLO‐GFP transgenic plants

For stable expression, *pHSP*::GFP, *pHSP*::PHYSCODILLO1‐GFP and *pHSP*::PHYSCODILLO2‐GFP were transformed into wild‐type protoplasts and successful integration confirmed following two rounds of G418 selection. Full details of construct generation are given in Methods S1.

### Protein expression analysis

For protein gel analysis, *P. patens* protonemal tissues expressing *pHSP*::GFP, *pHSP*::PHYSCODILLO1‐GFP and *pHSP*::PHYSCODILLO2‐GFP were harvested into liquid BCD protonemal medium and incubated at 22 (control), 34 or 38°C for 1 h before returning to room temperature. For MG132 experiments, tissue in liquid BCD was pretreated with 50 μM MG132 for 1 h before heat shock. Samples were collected at different times postinduction and flash‐frozen in liquid nitrogen before extraction. Sodium dodecyl sulfate–polyacrylamide gel electrophoresis (SDS‐PAGE) and western blotting were carried out using standard procedures as in Nibau *et al*. (2011).

For microscopy analysis, tissues were grown on BCD medium under standard growth conditions, and protein expression was induced for 1 h at 38°C before returning to 22°C for different lengths of time. For MG132 experiments, tissue in liquid BCD was pretreated with 50 μM MG132 for 1 h before heat shock. Confocal images were captured using a Leica SP2 inverted confocal microscope. Other images were captured using a Nikon SMZ1000 dissecting microscope and nis‐elements software (Nikon, Tokyo, Japan).

### Construction of *physcodillo1a/1b/2* triple deletion mutants and screening procedure

The *PHYSCODILLO1A/1B* double deletion construct was generated by cloning 5′ and 3′ homologous flanking sequences from *P. patens* genomic DNA and inserting them into the pMBL10a vector either side of a G418 resistance cassette (see Fig. S3a later). The resulting construct was transformed into *physcodillo2* mutant protoplasts (Moody *et al*., 2012). Two rounds of G418 selection were carried out to identify putative transformants. To verify the presence of a G418 resistance cassette within the *PHYSCODILLO1A/1B* locus and confirm the generation of *physcodillo1a/1b/2* triple deletion mutants, PCR was carried out using GoTaq DNA Polymerase (Promega). 5′ integration was confirmed using the primers P1 + 3KO5′F and G418.R.319 and 3′ integration was confirmed using the primers P1 + 3KO3′R and G418.F.341. PCR products were sequenced (see Fig. S3 later) and RT‐PCR was carried out to confirm loss of *PHYSCODILLO1* mRNA expression. Primer sequences are detailed in Table S1.

### Confirmation of *physcodillo1a/1b/2* triple deletion mutant by RT‐PCR

RNA and cDNA were prepared from 7‐d‐old protonemata (wild‐type, *physcodillo2*,* physcodillo1a/1b/2‐8* and *physcodillo1a/1b/2‐16*). Gene‐specific cDNA products were amplified using GoTaq DNA Polymerase (Promega). *PHYSCODILLO1* was amplified using P1‐RT.GSP.F and P1‐RT.GSP.R, *PHYSCODILLO2* was amplified using P2‐RT.GSP.F and P2‐RT.GSP.R and a positive control tubulin cDNA was amplified using PptubF and PptubR.

### *Physcomitrella patens* spore germination assays and protonemal area measurement

Sporangia were sterilized in 20% Parozone bleach for 15 min at room temperature and then washed four times in sterile water. Spores were released from sporangia by perforating them using a sterile pipette tip in a final volume of 1 ml of sterile water. Spore suspension (200 μl) was spread onto each of five Petri dishes containing cellophane‐overlaid spore regeneration medium. Spores were allowed to germinate at 22°C in long days (16 h : 8 h, light : dark). The percentage of germinating spores was calculated at regular intervals until all of the control spores had fully germinated. For each data point, > 200 spores were counted on each of three plates and the mean percentage germination ± SE of the mean was calculated. Each experiment was repeated multiple times. Plants were photographed on a Nikon SMZ1000 dissecting microscope, and the protonemal area was imaged and measured using Nikon's nis‐elements software package.

### Construction of *arabidillo* mutant rescue lines

35S::PHYSCODILLO1‐GFP and 35S::SELAGIDILLO‐GFP fusions were made in pGreen0029 (Hellens *et al*., 2000) by replacing ARABIDILLO1 in the 35S::ARABIDILLO1‐GFP construct (Coates *et al*., 2006). Full details of construct generation are given in Methods S1. Both constructs were transformed into *Agrobacterium tumefaciens* GV3101 with pSoup (Hellens *et al*., 2000) and transformed into *A. thaliana* by floral dip (Clough & Bent, 1998).

### Root assays

LR assays were carried out as in Coates *et al*. (2006). Seedling LR density was defined as the number of emerged LRs cm^−1^ of primary root. A minimum of 50 roots were assayed for each genotype in a single experiment and each experiment was repeated multiple times.

### *Arabidopsis thaliana* seed germination assays

Freshly harvested seeds from wild‐type, mutant and transgenic plants were surface‐sterilized in 5% (v/v) bleach for 5 min then washed with sterile water before plating (three to four replicates; *n *=* *50) onto water agarose (1%) supplemented with relevant concentrations of ABA (Sigma). After 4 d of chilling, seeds were incubated at 22°C under continuous light for 7 d, and germination was assessed as endosperm rupture by the radicle. Each assay was repeated multiple times.

## Results

**Figure 1:**
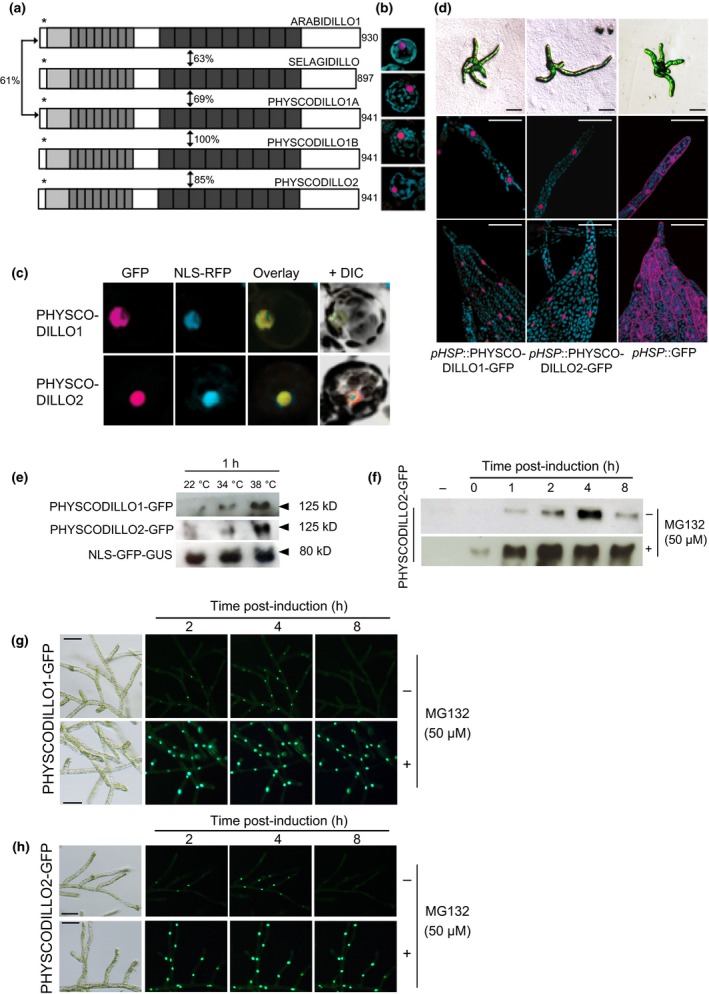
ARABIDILLO protein homologues in *Physcomitrella patens* and *Selaginella moellendorffii*. (a) ARABIDILLO homologues in *Arabidopsis thaliana* (ARABIDILLO1), *S. moellendorffii* (SELAGIDILLO) and *P. patens* (PHYSCODILLO1A, ‐1B and ‐2). Light grey boxes, F‐box; mid‐grey boxes, leucine‐rich repeats; dark grey boxes, Armadillo repeats. Asterisks, nuclear localization signals. Percentages indicate the degree of amino acid identity between the protein homologues, as indicated in Moody *et al*. (2012). Numbers at the C‐termini indicate amino acid length. (b) Constitutive expression of full‐length GFP‐tagged proteins in *P. patens* protoplasts. From the top, *35S*::ARABIDILLO1‐GFP;* 35S*::SELAGIDILLO‐GFP;*pUBI*::PHYSCODILLO1‐GFP;*pUBI*::PHYSCODILLO2‐GFP. Each image is representative of 15–20 protoplasts. (c) Protoplasts co‐transformed with *pUBI*::PHYSCODILLO1‐GFP or *pUBI*::PHYSCDILLO2‐GFP and a nuclear localisation signal‐red fluorescent protein (NLS‐RFP) nuclear marker. (d) Inducible expression of full‐length green fluorescent protein (GFP)‐tagged proteins in *P. patens* transgenic lines. From left to right, *pHSP*::PHYSCODILLO1‐GFP;*pHSP*::PHYSCODILLO2‐GFP;*pHSP*::GFP. Tissues from upper panels to lower panels: protonemal filaments (low magnification); protonemal filaments (confocal sections); leafy gametophore tissue (confocal sections). Bars, 50 μm. (e) Western blot showing inducible expression of *pHSP*::PHYSCODILLO1‐GFP and *pHSP*::PHYSCODILLO2‐GFP compared with a constitutively expressed NLS‐GFP‐GUS control (β‐glucuronidase) (Bezanilla *et al*., 2005). (f) Western blot showing degradation of heat‐shock‐induced PHYSCODILLO2‐GFP and its stabilization in the presence of the proteasome inhibitor MG132. (g) Fluorescence micrographs showing degradation of heat‐shock‐induced PHYSCODILLO1‐GFP and its stabilization in the presence of the proteasome inhibitor MG132. Bars, 50 μm. (h) Fluorescence micrographs showing degradation of heat‐shock‐induced PHYSCODILLO2‐GFP and its stabilization in the presence of the proteasome inhibitor MG132, similarly to PHYSCODILLO1‐GFP (compare with g). Bars, 50 μm.

*Arabidopsis thaliana* ARABIDILLO proteins localize to the nucleus, where they exert their control of LR development through physically interacting with flowering plant‐specific MYB transcription factors (Coates *et al*., 2006; Nibau *et al*., 2011; Gibbs *et al*., 2014). However, ARABIDILLO homologues with a high degree of protein identity to one another also exist in early‐diverging land plants, which lack both LRs and relevant MYB homologues (Nibau *et al*., 2011; Moody *et al*., 2012; Gibbs *et al*., 2014; Fig. 1a). To analyse the behaviour of ARABIDILLO proteins in an early‐diverging land plant, we examined the localization of ARABIDILLO homologues in transiently transformed *P. patens* protoplasts using a series of fusion proteins with green fluorescent protein (GFP) directly fused in‐frame to the C‐terminus of ARABIDILLO homologues, driven from constitutive promoters (Fig. 1a). ARABIDILLO1‐GFP, PHYSCODILLO1A/1B‐GFP (which are identical to one another (Moody *et al*., 2012) and subsequently are collectively referred to as PHYSCODILLO1), PHYSCODILLO2‐GFP and SELAGIDILLO‐GFP (the *S. moellendorffii* ARABIDILLO homologue; Moody *et al*., 2012) all show considerable sequence identity across the entire length of the protein (Fig. 1a). All the proteins localize to the nucleus (Fig. 1b): this was confirmed using a red fluorescent protein marker tagged with a nuclear localization signal (NLS‐RFP; Fig. 1c), which colocalized with the PHYSCODILLO‐GFP signal.

**Figure 2:**
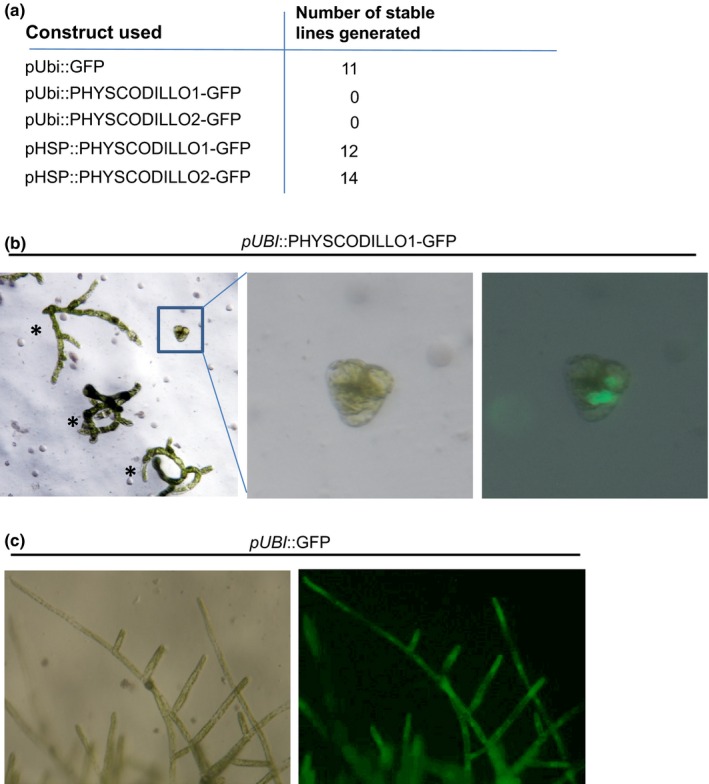
Constitutive expression of PHYSCODILLO proteins in *Physcomitrella patens* leads to arrested protoplast regeneration. (a) Numbers of stable transgenic lines generated as a result of transforming *P. patens* protoplasts with constructs encoding combinations of proteins (GFP or PHYSCODILLOs) driven from the constitutive maize *UBIQUITIN* promoter (*pUBI*) or the inducible heat‐shock promoter (*pHSP*). Using the constitutive promoter (*pUBI*) to drive PHYSCODILLO‐GFP fusion proteins results in no recovery of stable transgenic lines. *pUBI* driving green fluorescent protein (GFP) alone, or *pHSP* driving PHYSCODILLO‐GFP fusions leads to normal transgenic line generation. (b) Transient transformation of *P. patens* protoplasts with *pUBI*::PHYSCODILLO1‐GFP leads to the formation of small, arrested GFP‐expressing colonies that show no further cell growth (zoomed image) compared with untransformed protoplast regenerating colonies (asterisks). (c) A control *pUBI*::GFP fusion protein is not lethal, and mature regenerated transformants are obtained.

To further examine the spatial and temporal localization and behaviour of PHYSCODILLO proteins, we attempted to generate stable transgenic lines expressing GFP‐tagged PHYSCODILLO proteins under the control of the constitutive maize (*Zea mays*) *Ubiquitin‐1* promoter (*pUBI*). However, we found that overexpression of PHYSCODILLO‐GFP in protoplasts was toxic, inhibiting the outgrowth of protonemal filaments and preventing protoplast regeneration, so no transformants could be recovered (Fig. 2). This is in accordance with the data of Nibau *et al*. (2011), showing the possible toxicity of overexpressed ARABIDILLO protein in *A. thaliana*. To circumvent this problem, we generated stable transgenic *P. patens* lines where inducible expression of PHYSCODILLO1 or ‐2 fused to GFP was driven from the soybean (*Glycine max*) heat‐shock promoter (*pHSP*; Saidi *et al*., 2005) and induced at different stages of *P. patens* development. In both filamentous and leafy tissue, PHYSCODILLO‐GFP is detected in the nucleus, while GFP alone localizes to both nucleus and cytosol (Fig. 1d). We determined that good induction of PHYSCODILLO‐GFP expression occurs after just 1 h at 38°C (Figs 1e, S1).

ARABIDILLOs are unstable proteins that are turned over by the proteasome (Nibau *et al*., 2011). Using the inducible PHYSCODILLO lines, we were able to show by confocal microscopy and western blotting that PHYSCODILLO proteins are also turned over by the proteasome (Figs 1f–h, S1), as, like ARABIDILLOs, they are stabilized by the proteasome inhibitor MG132 (Nibau *et al*., 2011). This fits with the previous observation that the key regions required for ARABIDILLO/PHYSCODILLO instability, namely the F‐box and leucine‐rich repeat regions, are highly conserved (Nibau *et al*., 2011). This demonstrates similar protein characteristics for PHYSCODILLOs and ARABIDILLOs despite *c*. 420 million yr of evolutionary divergence (Hedges *et al*., 2006).

**Figure 3:**
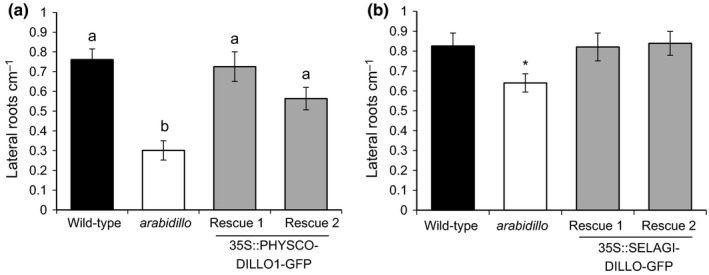
PHYSCODILLO and SELAGIDILLO can both rescue the *Arabidopsis thaliana arabidillo1/2* mutant lateral root phenotype. (a) Mean lateral root density measured 8 d after germination for wild‐type (black bar), *arabidillo1/2* mutant (white bar) and two independent *arabidillo1/2* mutant lines constitutively expressing PHYSCODILLO1‐GFP from the 35S promoter (Rescue 1 and Rescue 2, grey bars). One‐way ANOVA shows significant differences between genotypes (*P *<* *0.0001). A Tukey *post hoc* test shows that the *arabidillo* mutant is significantly different from wild‐type (*P *<* *0.01) and from Rescue 1 (*P *<* *0.01) and Rescue 2 (*P *<* *0.05). Different lowercase letters indicate significant differences between genotypes. Error bars show ± SE of the mean. (b) Mean lateral root density 8 d after germination for wild‐type (black bar), *arabidillo1/2* mutant (white bar) and two independent *arabidillo1/2* mutant lines constitutively expressing SELAGIDILLO‐GFP from the 35S promoter (Rescue 1 and Rescue 2, grey bars). One‐way ANOVA indicated that differences between genotypes were not quite significant (*P *=* *0.057), although pairwise *t*‐tests comparing the mutant and rescue lines with the wild‐type indicated that the wild‐type was different from the mutant (*, *P *<* *0.05) but not different from either rescue line. Error bars show ± SE of the mean.

To further investigate PHYSCODILLO behaviour and function, we investigated whether *P. patens* and *S. moellendorffii* ARABIDILLO homologues were able to substitute for *A. thaliana* ARABIDILLO function during LR formation. We generated stable transgenic *A. thaliana* lines expressing either PHYSCODILLO1 or SELAGIDILLO1 driven by the cauliflower mosaic virus (CaMV) 35S promoter, and found that both bryophyte and lycophyte ARABIDILLO homologues were able to complement the reduced LR phenotype of the *arabidillo1/2* mutant (Fig. 3a,b), despite the facts that *P. patens* has no multicellular rooting structures, and that *S. moellendorffii* has tip‐bifurcating roots (Banks, 2009; Jones & Dolan, 2012). Thus, early‐diverging ARABIDILLO homologues had the capacity to affect LR development before the evolution of true, branched rooting structures occurred, suggesting that they were functionally co‐opted into pathways regulating novel structures that arose during the evolution of flowering plants.

**Figure 4:**
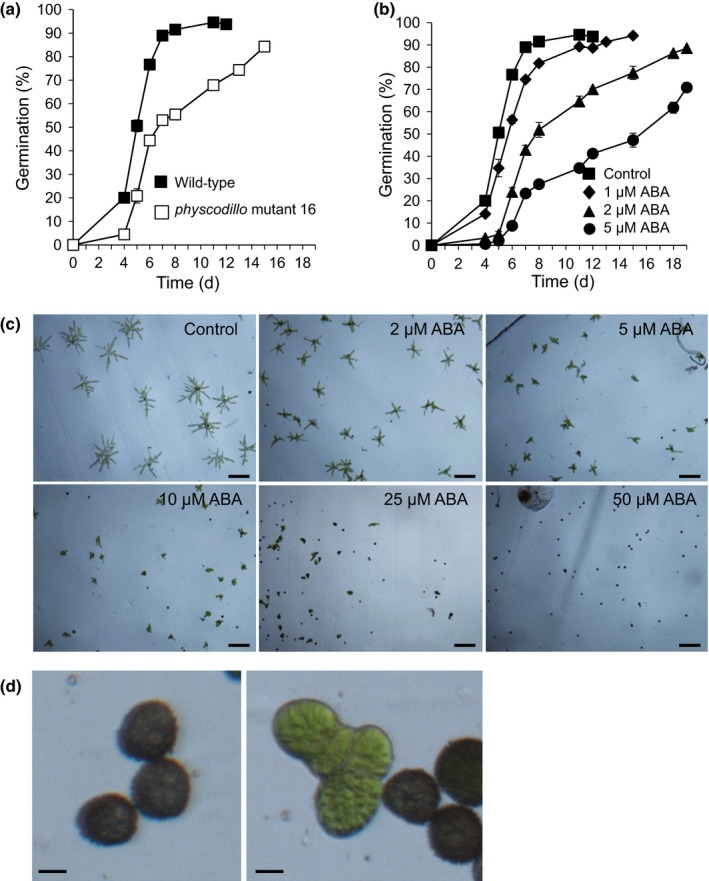
*Physcomitrella patens physcodillo* triple mutants show slower germination, and abscisic acid (ABA) inhibits *P. patens* spore germination. (a) Germination of wild‐type and *physcodillo* mutant spores. A Mann–Whitney test did not identify significant differences between genotypes (*P *=* *0.08) although *z*‐tests suggested highly significant (*P *<* *0.01) differences on all of days 4, 5, 6, 7, 8 and 11. (b) Inhibition of *P. patens* spore germination by 1–5 μM ABA. Error bars show ± SE of the mean. A Kruskal–Wallis test for differences between treatments was significant (*P *<* *0.05) on days 4, 5, 6, 7, 8 and 12. A Dunn's test identified significant (*P *<* *0.05) differences between control and 2 μM ABA and highly significant (*P *<* *0.01) differences between control and 5 μM ABA on days 4, 5, 6 and 7. (c) Effect of 0–50 μM ABA on germination in a population of spores. More ungerminated spores are seen as ABA concentration increases, in addition to a reduction in plant size (cell length) occurring. Bars, 100 μm. (d) Higher magnification images of spores treated with 25 μM ABA. The left‐hand panel shows ungerminated spores, while the right‐hand panel shows a small plant on the left (with shortened cells, from a germinated spore) and ungerminated spores on the right. Bar, 10 μm.

We previously showed that a single *PHYSCODILLO2* knock‐out in moss has no obvious phenotype, suggesting that the homologues function redundantly (Moody *et al*., 2012), similarly to what is observed in *A. thaliana* (Coates *et al*., 2006). To investigate the function(s) of PHYSCODILLO proteins in *P. patens*, we therefore generated two independent triple *physcodillo1a/1b/2* loss‐of‐function mutants by targeted gene replacement, deleting the entire 23‐kb *PHYSCODILLO1A/1B* locus in a *physcodillo2* mutant background (Moody *et al*., 2012; Figs S2a,b, S3). The replaced locus was confirmed by sequencing across the insertion site (Fig. S3). Using RT‐PCR we confirmed the absence of *PHYSCODILLO* mRNAs in these knockout lines (Fig. S2c). Similarly to the *physcodillo2* mutants (Moody *et al*., 2012), *physcodillo1a/1b/2* mutant plants are overall morphologically similar to wild‐type, producing chloronema, caulonema, gametophores with rhizoids (Fig. S4a,b), antheridia, archegonia and sporophytes in a similar time frame. However, we noticed that the spores of *physcodillo1a/1b/2* mutants germinated more slowly than those of wild‐type, suggesting a potential role for PHYSCODILLOs in regulating this process (Fig. 4a).

In seed plants, ABA is a key inhibitor of germination and promotes seed dormancy (Finkelstein *et al*., 2008; Holdsworth *et al*., 2008). Some evidence also implicates ABA in the regulation of fern spore germination (Singh *et al*., 1990; Yao *et al*., 2008). However, neither the process of *P. patens* spore germination nor the effects of ABA on spores have previously been studied in detail (Glime, 2015). We showed a dose‐dependent inhibition of the wild‐type *P. patens* spore germination rate by ABA (Fig. 4b–d). This suggests that ABA has a similar negative regulatory role in both spore and seed germination, despite the different developmental origins of spores and seeds. We showed that *P. patens* spores showed much lower sensitivity to ABA than *A. thaliana* seeds, as 5 μM ABA, which would completely inhibit *A. thaliana* germination, significantly reduced the spore germination rate without inhibiting germination completely (Fig. 4b,c).

**Figure 5:**
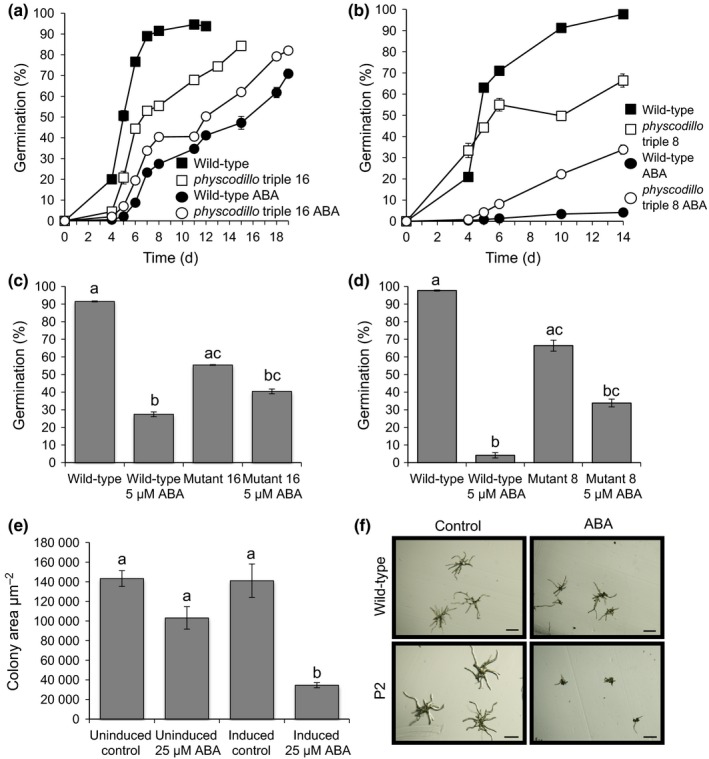
*Physcomitrella patens physcodillo* triple mutants show insensitivity to the inhibitory effects of abscisic acid (ABA) during germination while a *P. patens* PHYSCODILLO‐overexpressing line shows ABA hypersensitivity during early growth. (a) Germination of wild‐type and *physcodillo* triple mutant line 16 spores on medium containing 5 μM ABA or solvent‐only control. Kruskal–Wallis tests showed significant (*P *<* *0.05) differences between genotypes/treatments on days 4, 5, 6, 7, 8 and 11. Dunn's tests showed significant (*P *<* *0.01) differences between wild‐type spores with and without ABA on days 4, 5, 6, 7, 8 and 11, significant (*P *<* *0.05) differences between ABA‐treated wild‐type and untreated *physcodillo* mutants on days 5, 6, 7, 8 and 11 and significant (*P *<* *0.05) differences between wild‐type and ABA‐treated *physcodillo* mutants on days 5, 6, 7, 8 and 11. (b) Germination of wild‐type and *physcodillo* triple mutant line 8 mutant spores germinated on medium containing 5 μM ABA or solvent‐only control. Kruskal–Wallis tests showed significant (*P *<* *0.05) differences between genotypes/treatments on days 4, 5, 6, 10 and 14. Dunn's tests showed significant (*P *<* *0.01) differences between wild‐type spores with and without ABA on all days and significant (*P *<* *0.05) differences between ABA‐treated wild‐type and untreated *physcodillo* mutants on all days. (c) Insensitivity of *physcodillo* triple mutant 16 to 5 μM ABA. Data are shown for day 8. A Kruskal–Wallis test for differences between genotypes and treatments was significant (*P *<* *0.05). Dunn's test identified differences between groups: a and b, *P *<* *0.001; b and c, *P *<* *0.05; a and c, *P *<* *0.05. Error bars show ± SE of the mean. (d) Insensitivity of *physcodillo* triple mutant 8 to 5 μM ABA. Data are shown for day 14. A Kruskal–Wallis test for differences between genotypes and treatments was significant (*P *<* *0.05). Dunn's test identified differences between groups: a and b, *P *<* *0.001; b and c, *P *<* *0.05; a and c, *P *<* *0.05. Error bars show ± SE of the mean. (e) Transgenic *pHSP*::PHYSCODILLO2‐GFP plants were either grown at 22°C on medium containing either solvent control or 25 μM ABA (left‐hand bars), or exposed to daily 1‐h heat shock and grown on medium containing either solvent control or ABA (right‐hand bars). One‐way ANOVA showed significant differences between treatments, with a Tukey's *post hoc* test showing that only plants with induced PHYSCODILLO2‐GFP expression grown on ABA (b) were significantly different (*P *<* *0.05) from those in other treatments (a). Error bars show ± SE of the mean. (f) Representative morphology of wild‐type vs heat‐shocked transgenic *pHSP*::PHYSCODILLO2‐GFP plants germinated from spores and grown on solvent control or 25 μM ABA. Bars, 100 μm.

We also examined the response of *physcodillo1a/1b/2* mutants, and found that both *physcodillo1a/1b/2* mutant alleles are less sensitive to the ABA‐mediated inhibition of the spore germination rate than wild‐type, implying that PHYSCODILLOs play a role in regulating the response to ABA during this process (Fig. 5a–d). When we examined other known ABA‐mediated processes, namely response to desiccation/drought and freezing, we found that the *physcodillo1a/1b/2* plants showed no obvious differences in their desiccation or freezing tolerance compared with wild‐type plants (Fig. S4c,d), indicating a specific role in germination.

To extend these findings, we examined the effects of PHYSCODILLO overexpression on ABA sensitivity in *P. patens*. Because of the lethality of constitutive PHYSCODILLO overexpression, we used heat‐shock‐inducible lines. We showed that heat‐shock‐inducible overexpression of the PHYSCODILLO2 protein can lead to ABA hypersensitivity during early growth, when spores are germinated in the presence of ABA (Fig. 5e,f). An inducible transgenic line expressing the β‐glucuronidase (GUS) protein from the heat‐shock promoter showed no such hypersensitivity, linking the observed phenotype to PHYSCODILLO overexpression (data not shown).

**Figure 6:**
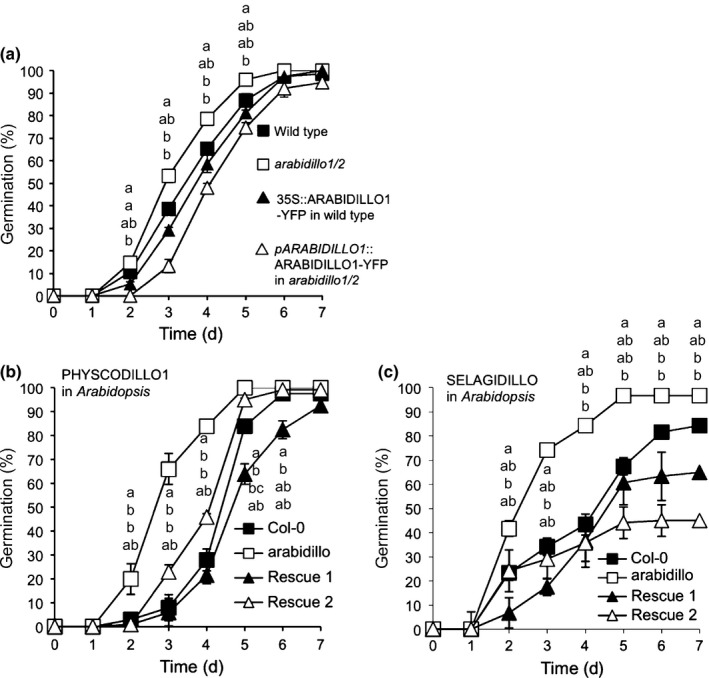
Abscisic acid (ABA) insensitivity of the *Arabidopsis thaliana arabidillo* mutant during seed germination and its rescue by both PHYSCODILLO and SELAGIDILLO proteins. (a) Seeds from wild‐type (Col‐0), *arabidillo* mutant, a 35S::ARABIDILLO‐YFP overexpressing line (Coates *et al*., 2006) and an *arabidillo* mutant stably expressing *pARABIDILLO1*::ARABIDILLO1‐YFP (Coates *et al*., 2006; Nibau *et al*., 2011) plated on 1 μM ABA with percentage germination assayed over 7 d. Error bars show ± SE of the mean. A Kruskal–Wallis test identified significant (*P *<* *0.05) differences between genotypes on days 2, 3, 4 and 5. Different letters indicate significant (*P *<* *0.05) differences between genotypes determined using Dunn's test for, top to bottom, *arabidillo1/2*, wild‐type, 35S::ARABIDILLO1‐YFP in wild‐type, and pARABIDILLO1::ARABIDILLO1‐YFP in *arabidillo1/2*. (b) Seeds from wild‐type, *arabidillo* mutant, and *arabidillo* mutant stably expressing PHYSCODILLO1 (*35S*::PHYSCODILLO1‐GFP; two independent lines, Rescue 1 and Rescue 2) plated on 1 μM ABA with percentage germination assayed over 7 d. A Kruskal–Wallis test identified significant differences (*P *<* *0.05) between genotypes on days 2, 3, 4, 5 and 6. Different letters indicate significant differences between genotypes determined using Dunn's test (*P *<* *0.05) for data points, from top to bottom, for *arabidillo1/2*, wild‐type, Rescue 1 and Rescue 2. (c) Seeds from wild‐type, *arabidillo* mutant, and *arabidillo* mutant stably expressing SELAGIDILLO (35S::SELAGIDILLO‐GFP: two independent lines, Rescue 1 and Rescue 2) plated on 1 μM ABA with percentage germination assayed over 7 d. A Kruskal–Wallis test identified significant differences (*P *<* *0.05) between genotypes on days 2, 3, not 4 (*P *=* *0.07), 5, 6 and 7. Different letters indicate significant differences between genotypes determined using Dunn's test (*P < *0.05) for data points, from top to bottom, for *arabidillo1/2*, wild‐type, Rescue 1 and Rescue 2.

We previously showed that *arabidillo1/2* mutants in *A. thaliana* do not have altered sensitivities to ABA with regard to LR development (Nibau *et al*., 2011); however, we did not investigate other potential ABA functions for ARABIDILLOs in *A. thaliana*. To determine whether the role of PHYSCODILLOs in regulating ABA responses during germination is conserved, we examined seed germination in *A. thaliana*. Remarkably, we found that the *A. thaliana arabidillo1/2* mutant is relatively insensitive to the ABA‐mediated inhibition of seed germination compared with the wild‐type. Moreover, ARABIDILLO1‐overexpressing and ‘rescue’ lines displayed an opposite ABA‐hypersensitive phenotype (Fig. 6a). Both mutant and overexpressing seeds germinated as wild‐type in the absence of ABA (data not shown). These data imply a conserved role for ARABIDILLO homologues in ABA‐regulated germination across land plants, as well as a unique role in LR development in flowering plants (Coates *et al*., 2006; Gibbs *et al*., 2014). To further investigate this conserved germination function, we asked whether the early‐diverging *P. patens* and *S. moellendorffii* ARABIDILLO homologues could rescue the *A. thaliana arabidillo1/2* mutant germination phenotype. Reintroduction of either PHYSCODILLO or SELAGIDILLO into the *arabidillo* mutant led to a complementation of the ABA‐insensitive germination phenotype (Fig. 6b,c). Therefore, despite the different developmental origins of spores and seeds, *P. patens* and *S. moellendorffii* ARABIDILLO homologues can replace ARABIDILLOs during *A. thaliana* germination, demonstrating a novel and evolutionarily ancient function for the ARABIDILLO protein family regulating the germination of desiccation‐resistant dispersal units in response to ABA.

## Discussion

The production of dispersal units such as spores and seeds was a critical step enabling plant survival and movement on land. Germination of such structures is tightly regulated to ensure that plants establish themselves in the right place and at the right time under favourable conditions (Holdsworth *et al*., 2008). In flowering plants and gymnosperms, seeds are multicellular structures that protect the diploid embryo, which gives rise to the subsequent sporophyte generation. In bryophytes, unicellular spores arise by meiosis and give rise to the haploid gametophyte, and are therefore of different developmental origin from seeds (Pires & Dolan, 2012).

Our studies reveal for the first time that the hormone ABA has evolved to regulate functionally equivalent germination processes in spore‐ and seed‐producing plants.

Furthermore, we identify conserved PHYSCODILLO/ARABIDILLO proteins as novel modulators of the germination of land plant desiccation‐resistant dispersal units in conjunction with ABA. We suggest that PHYSCODILLO/ARABIDILLO proteins represent a conserved node in a germination‐regulatory network that includes ABA, in both seeds and spores.

ABA has ancient evolutionary origins, being produced in cyanobacteria and all major eukaryote lineages: shared roles for ABA in stress tolerance have been proposed across these taxa (Takezawa *et al*., 2011, 2015). ABA‐mediated stomatal control has ancient origins (Chater *et al*., 2011; Ruszala *et al*., 2011; Lind *et al*., 2015) and ABA has also been implicated in developmental transitions in land plants, green algae and the Apicomplexan *Toxoplasma* (Takezawa *et al*., 2011).

Although ABA is widespread throughout taxa, land plants probably acquired unique ABA signalling mechanisms early during their history (Hauser *et al*., 2011), which have subsequently been conserved in terms of changes in gene expression (Knight *et al*., 1995; Kamisugi & Cuming, 2005; Cuming *et al*., 2007; Wang *et al*., 2010). ABA responses are evolutionarily conserved and have been co‐opted into both gametophyte and sporophyte tissues: ABA signalling regulates stress tolerance in the vegetative tissues of bryophytes (Cuming *et al*., 2007; Komatsu *et al*., 2009, 2013; Khandelwal *et al*., 2010; Richardt *et al*., 2010; Tougane *et al*., 2010) as well as stomatal opening in the sporophyte (Chater *et al*., 2011). ABA is well known for mediating stress responses, stomatal opening and germination in seed plants (Nambara & Marion‐Poll, 2005; Christmann *et al*., 2006; Wasilewska *et al*., 2008).

It is tempting to speculate that ARABIDILLO and PHYSCODILLO proteins have conserved protein interactors and/or transcriptional targets in spore and seed germination, especially given their highly conserved sequences, domain structure and proteasomal regulation (Nibau *et al*., 2011; Fig. 1). Interestingly, in charophytes, ABA can inhibit germination in light‐treated oospores (Takatori & Imahori, 1971). Whether charophyte ARABIDILLO homologues (which our searches suggest exist; Timme & Delwiche, 2010; Hori *et al*., 2014; Matasci *et al*., 2014; Wickett *et al*., 2014) are part of this regulation is currently unknown. We also propose that ARABIDILLOs acquired a novel ABA‐independent function in flowering plants, regulating LR development, via interaction with flowering plant‐specific MYB transcription factors (Gibbs & Coates, 2014; Gibbs *et al*., 2014).

Our work reveals a retention of ancient functions for plant ARABIDILLO proteins, despite them evolving to have novel additional roles in flowering plants. Furthermore, we have added a new dimension to the land plant ABA story, demonstrating the first conservation of molecular mechanisms between spore and seed germination.

## Author contributions

J.C.C., L.A.M. and Y.S. conceived the experiments; L.A.M., Y.S., D.J.G., A.C., D.H., E.F.V., K.K.B., S.J.B. and J.C.C. performed the experiments; L.A.M., Y.S., D.J.G., D.H. and J.C.C. analysed data; L.A.M., Y.S., D.J.G. and J.C.C. wrote the paper.

## Supporting information

Please note: Wiley Blackwell are not responsible for the content or functionality of any supporting information supplied by the authors. Any queries (other than missing material) should be directed to the *New Phytologist* Central Office.

**Fig. S1** PHYSCODILLO‐GFP fusions are turned over by the proteasome.
**Fig. S2** Generation of a *physcodillo1A/1B/2* triple mutant by targeted gene replacement.
**Fig. S3** Sequencing of the replaced *physcodillo1A/1B* locus in the *physcodillo2* mutant background, generating triple mutants number 8 and number 16.
**Fig. S4** *physcodillo* triple mutants show no obvious differences in rhizoid development or vegetative ABA responses.
**Table S1** Primers used to generate and characterize *PHYSCODILLO1A/1B* gene replacement and generate *arabidillo* mutant rescue lines
**Methods S1** Generation of PHYSCODILLO‐GFP transgenic plants, construction of *arabidillo* mutant rescue lines, construction of *physcodillo1A/1B/2* triple deletion mutants and screening procedure.
