# Supplementary material for: An ancient and conserved function for Armadillo‐related proteins in the control of spore and seed germination by abscisic acid
Source: New Phytol. 2016 Apr 4;211(3):940–51. doi: 10.1111/nph.13938 (PMC4982054; doi:10.1111/nph.13938)
Supplement: Supplementary file 1 — Fig. S1 PHYSCODILLO‐GFP fusions are turned over by the proteasome. Fig. S2 Generation of a physcodillo1A/1B/2 triple mutant by targeted gene replacement. Fig. S3 Sequencing of the replaced physcodillo1A/1B locus in the physcodillo2 mutant background, generating triple mutants number 8 and number 16. Fig. S4 physcodillo triple mutants show no obvious differences in rhizoid development or vegetative ABA responses. Table S1 Primers used to generate and characterize PHYSCODILLO1A/1B gene replacement and generate arabidillo mutant rescue lines Methods S1 Generation of PHYSCODILLO‐GFP transgenic plants, construction of arabidillo mutant rescue lines, construction of physcodillo1A/1B/2 triple deletion mutants and screening procedure. [file NPH-211-940-s001.pdf]

New Phytologist

New Phytologist Supporting Information Figs S1–S4, Table S1 and Methods S1

Article title: An ancient and conserved function for Armadillo-related proteins in the control of seed and spore germination by abscisic acid

Authors: Laura A. Moody, Younousse Saidi, Daniel J. Gibbs, Anushree Choudhary, Daniel Holloway, Eleanor F. Vesty, Kiran Kaur Bansal, Susan J. Bradshaw and Juliet C. Coates

Article acceptance date: 15 February 2016

The following Supporting Information is available for this article:

Fig. S1 PHYSCODILLO-GFP fusions are turned over by the proteasome.

Fig. S2 Generation of a *physcodillo1A/1B/2* triple mutant by targeted gene replacement.

Fig. S3 Sequencing of the replaced *physcodillo1A/1B* locus in the *physcodillo2* mutant background, generating triple mutants number 8 and number 16.

Fig. S4 *physcodillo* triple mutants show no obvious differences in rhizoid development or vegetative ABA responses.

Table S1 Primers used to generate and characterize *PHYSCODILLO1A/1B* gene replacement and generate *arabidillo* mutant rescue lines

Methods S1 Generation of PHYSCODILLO-GFP transgenic plants, construction of *arabidillo* mutant rescue lines, construction of *physcodillo1a/1b/2* triple deletion mutants and screening procedure.

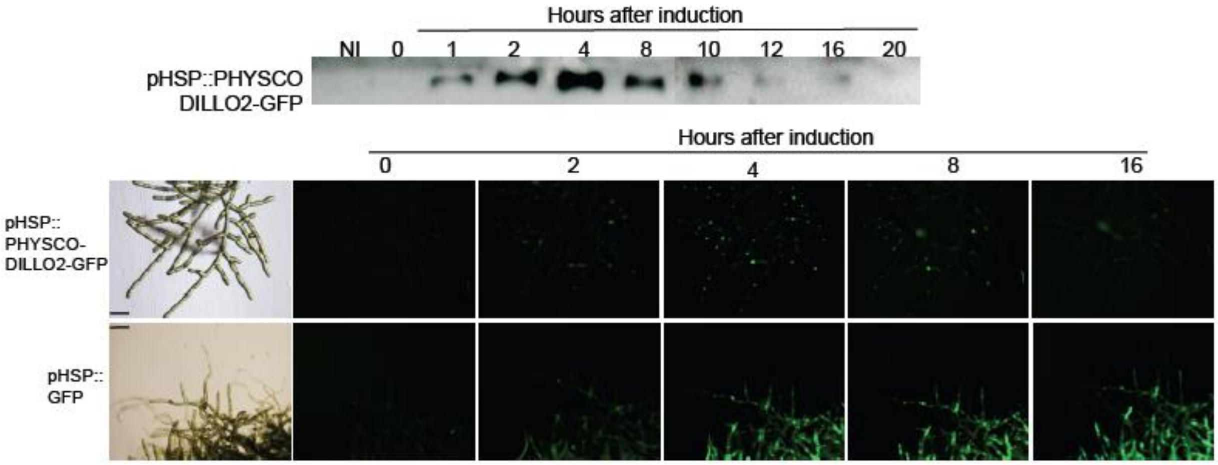

Fig. S1 PHYSCODILLO-GFP fusions are turned over by the proteasome. Stability of *pHSP*::PHYSCODILLO2-GFP over a longer time period: upper panel, Western blot with anti-GFP; lower panel, fluorescence compared to a *pHSP*::GFP control.

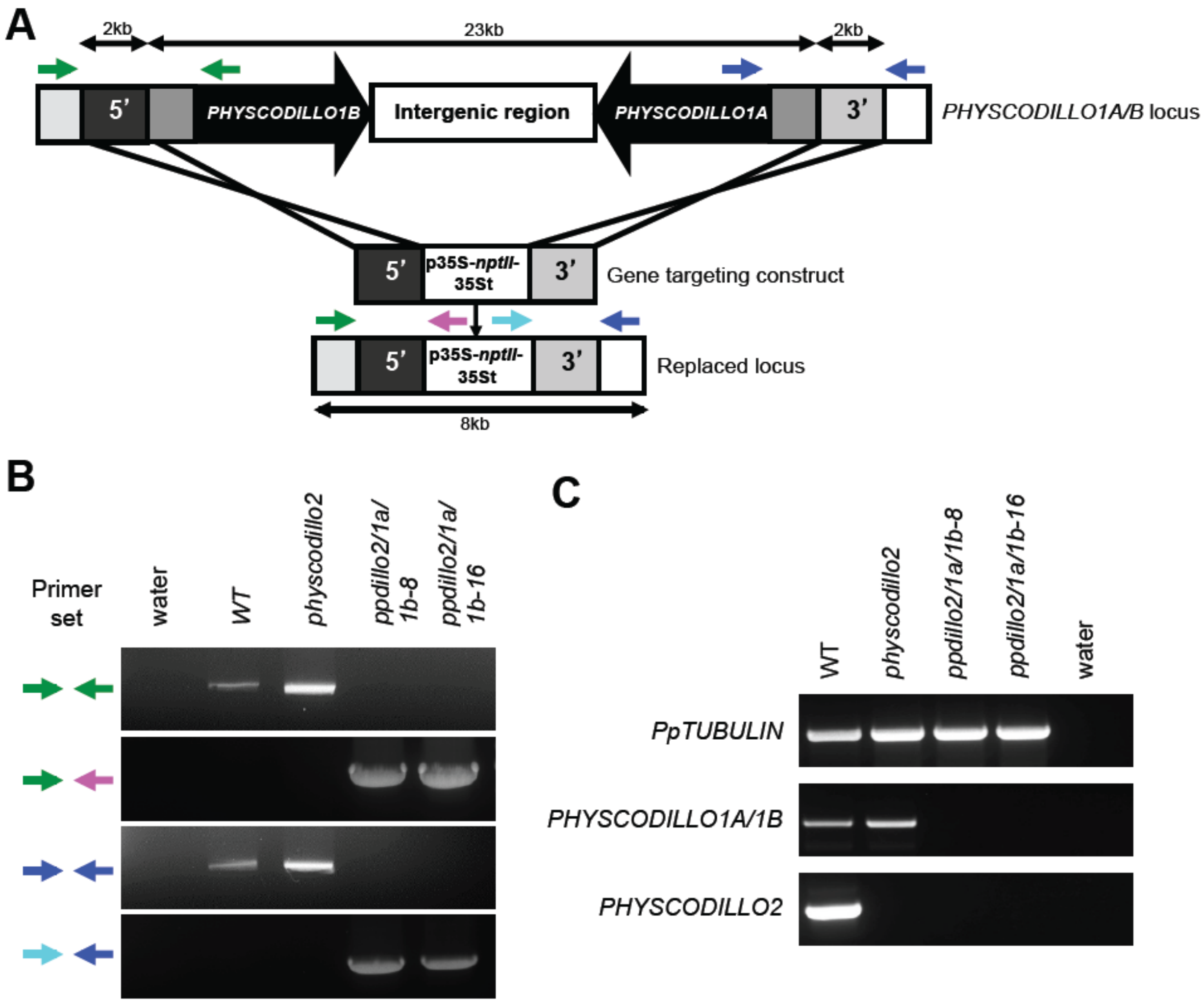

Fig. S2 Generation of a *physcodillo1A/1B/2* triple mutant by targeted gene replacement. (A) Strategy for replacing the entire 23kb *PHYSCODILLO1A/1B* locus with the *nptII* resistance cassette in the *physcodillo2* mutant (25) background. The primers indicated in green, blue, magenta and cyan were used on wild type and mutant genomic DNA to obtain the PCR products shown in (B). (B) Characterisation of the *physcodillo1A/1B/2* triple mutant lines. Green and blue primer pairs demonstrate the presence of the *PHYSCODILLO1A/1B* locus in wild type and *PHYSCODILLO2* mutant plants, and its absence in triple mutant 8 and 16. Green–magenta and cyan–blue primer pairs demonstrate insertion of the gene replacement cassette in the *physcodillo1A/1B/2* triple mutants 8 and 16. PCR products generated using magenta and cyan pairs were sequenced; full sequences are given in Fig. S4. (C) RT-PCR demonstrates the presence of *PHYSCODILLO1* gene expression in the wild type and *physcodillo2* mutant, but not

in two independent triple mutant lines (line 16 and line 8). Furthermore, *PHYSCODILLO2* expression is absent from both the *physcodillo2* and *physcodillo1A/1B/2 mutants* compared with the wild type control. *PpTUBULIN* was amplified as a positive control. Primer sequences are given in Table S1.

Fig. S3 Sequencing of the replaced *physcodillo1A/1B* locus in the *physcodillo2* mutant background, generating triple mutants number 8 and number 16. PCR products were amplified as outlined in the Materials and Methods section, using the primers detailed in Table S1. Colour coding indicates the different regions of the replaced locus, and this is indicated in each sequence.

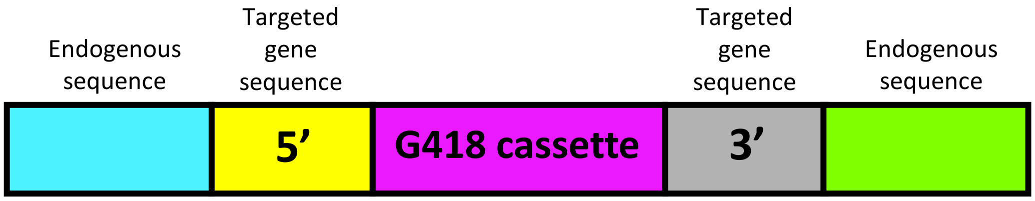

***physcodillo1A/1B/2*-line 8**
>5′ insertion PCR product sequence

```
GGAAANNNGCCCCTNAAAATTGTTGAGCTTGAGAGCTGGTGTGATGTGTTCGGGAGAGAGNAGAGACGAGAGTTTGAACGATCACGACGACTGGCTC
CGGTCGGAACGAGTGTTTGACAGTGGAAGGCTTGATCCAAATGTCTTGTGGCCTGAGTGATGTTTTCGTGGTTGAGGGCTTTTCAACTCCGCAGAGA
CAGTTGGTGCTGAAAAGTCTGGGGAAGCAAGTGGGTAGATTCGAATTTGTGCCGTTAGGTCTCCGTCGACACTGGAATTAGCGGTTACACGCCGAGT
GAACTTGTTAGTGAGCATTCGGACAGACCATGCCCTAGCAATCTCGCTCGGTTAGGCTCGTCATTTGGAAACGCTGAATCCGTCCGCAAAGCTTTCT
CTCTTTCTCCCACTACCCCTACTCGTAATTCTCTCCCCACACACCTTCATTTGGGCAGTATGGATGCCAATTTTCTTCCCGTTGACTACTTCAAATT
TCCGTACTCAAAGTGTCCTCCTGACCTGTGACGCGTTGCCGAAGATATTGCTGGCTGAATTCGGCGTCTCTCTGCAACGTAGGACAAATGCTTCAGA
GTGCCCGGTCAAAGTAATACGCAGATGGATATCCCGTTTCTTCAACACGAAATTTCAAACTAGCCACACGATAGTTCAAAACGGAGAAGTGAGAGGA
GAAAACGACACCACAACGGCTCATCTCGAAGTTTGCGAAGATCCGAAACAAAGAAGTTGGGGCATAGATATGAGGGGGCGCAGGATAGTGGATTCAA
AGTGATATCTGGATTGGAGCGATCAACTAGAGGCAACAGTCAGATTGATCATGGGACAAGAAACATGAGGTCATTATAAAACCAGTACCACTGAAAA
ATATTCAACGATTTTGCTTCTCAACTTCTGGAACAAGTTTGGAAAGTACGTAGTATAATCAATTAATGGGAGGGACAGCACATGAAATATCCAACAA
GCGGCGGCGCGAAGTCAATGTGGCGGTGGACGATCAGGAGCAGCAGCAGACTGTTGTCTATAAGAAAGCCAGGATAACATCGTCGCCGACGTCCGCT
TGTGGTGCATCGGCGGTGCCCCCTATAGAATCAGGTGTTAAAATTGGCGCGGGTCGCGATGCCCATTGGACAAGTCTGCCAGATGAAACCGTGCTTG
GGTTGTTTAATCTGCTGAACCACAGGGACATGGCGAGCCTTGCGTCCGTGTGAAAGGGATGGCAGGTGCTGGGGAGCTCGCAGTCGCTGTGGAATTC
GTCGGATCTTCGGNTGCACACGTTGAATTCGGAGATGGTGTCTGCGCTGGCAGGACAATGCTCGAATTTGGAGGCGCTGAAGTTCCGCAGAGGTGCC
TTTGCGAGCTCAATTGTGGGGTTGCAGGCGAAGGGGCTTAGGGAAATGAGCGGCGATTGCTGCAGCCAGCTGTCGGATGCAACCCTGTCCATGGTGG
TCGCCCGGCATGCGAACCTGGAGAGCAAAGGCCTTTTGAACTACCGATCTCTCTCCCCATCTCGATCTTCCTCCCTCTCGCTTTCGTGATTGATGTT
AATCCCAGAGAATAAAGTCCACCTGACCCAATTTTGTAAAACAACTGCTTTGAAAAGCTCCAGCATTCTCACACAGAATGAAGCATTGTGCGTGCTC
GCGGTCCCAACAAGCACTGGCGAGTTTACGGCGAAAAAGTTCCTGGCGCAGGTACAACGTGAAAAAGTAGGAGAGTGCAATTCACTACAGAGAACTT
TCCGAGCGAAGTCCATCAAATTTTGGGTTCTGGCACTGCCTCATCGCGACGAACGACGGTCCAACATGGTCAACGAAACCGAGAGAACAACTTTCTT
GTTCGTCATCAGAGCTTCATTTCCGTTCTTACCAGACGCCGTTCTAGGGCGAAGTCAACTCCAACAACAACAACAGCTTCCCTGAGATAAATGCCTT
CTAAAAGAGAGTACTATTACCAGCTTGTGACAGCCGAGAACATTAAGAATTATACTACTTTAATTTCTCGAATTTATCANAATNAGTCCAACAACAT
ATGCATAAAAATAAATATAAATCATGCATATGCATAAAAATAAATCCGGATCCCCCGGGCTGCAGGAATTCGATTCGAGGGGGGGCCCGGTACCCCT
ACTCCAAAAATGTCAAAGATACAGTCTCAGAAGACCAAAGGGCTATTGAGACTTTTCAACAAAGGGTAATTTCGGGAAACCTCCTCGGATTCCATTG
CCCAGCTATCTGTCACTTCATCGAAAGGACAGTAGAAAAGGAAGGTGGCTCCTACAAATGCCATCATTGCGATAAAGGAAAGGCTATCATTCAAGAT
GCCTCTGCCGACAGTGGTCCCAAAGATGGACCCCCACCCACGAGGAGCATCGTGGAAAAAGAAGACGTTCCAACCACGTCTTCAAAGCAAGTGGATT
GATGTGACATCTCCACTGACGTAAGGGATGACGCACAATCCCACTATCCTTCGCAAGACCCTTCCTCTATATAAGGAAGTTCATTTCATTTGGAGAG
GACAGCCCAAGCATGGAGAACCGACCTGCAGGCATGATTGAACAAGATGGATTGCACGCAGGTTCTCCGGCCGCTTGGGTGGAGAGGCTATTCGGCT
ATGACTGGGCACAACAGACAATCGGCTGCTCTGATGCCGCCGTGTTCCGGCTGTCAGCGCAGGGGCGCCCGGTTCTTTTTGTCAAGACCGACCTGTC
CGGTGCCCTGAATGAACTCCAAGACGAGGCAGCGCGGCTATCGTGGCTGGCCACGACGGGCGTTCCTTGCGCAGCTGTNCTCTAAANGGNCACTGAA
GCGGGAAGGGACTGGCTCCTATTGGCCTTTATTNAGGGGGCAGGATCTCCTGTCATCTCACCTTGCTCCTGCCGAGAAAGTTTCT
```

Endogenous (genomic, non-targeted) sequence
5′flanking (targeting) sequence
G418 cassette sequence

New Phytologist

***physcodillo1A/1B/2*-line 8**

>3′ insertion PCR product sequence

CCTTCTTGACGAGTTCTTCTGAGCGGGACTCTGGGGTTCGGACGGTACGCTGAAATCACCAGTCTCTCTCTACNAATCTATCTCTCTCTATTTTCTC
CATAAATAATGTGTGAGTAGTTTCCCGATAAGGGAAATTAGGGTTCTTATAGGGTTTCGCTCATGTGTTGAGCATATAAGAAACCCTTAGTATGTAT
TTGTATTTGTAAAATACTTCTATCAATAAAATTTCTAATTCCTAAAACCAAAATCCAGTACTAAAATCCAGATCTCCTAAAGTCCCTATAGATCTTT
GTCGTGAATATAAACCAGACACGAGACGACTAAACCTGGAGCCCAGACGCCGTTCGAAGCTAGAAGTACCGCTTAGGCAGGAGGCCGTTAGGGAAAA
GATGCTAAGGCAGGGTTGGTTACGTTGACTCCCCCGTAGGTTTGGTTTAAATATGATGAAGTGGACGGAAGGAAGGAGGAAGACAAGGAAGGATAAG
GTTGCAGGCCCTGTGCAAGGTAAGAAGATGGAAATTTGATAGAGGTACGCTACTATACTTATACTATACGCTAAGGGAATGCTTGTATTTATACCCT
ATACCCCCTAATAACCCCTTATCAATTTAAGAAATAATCCGCATAAGCCCCCGCTTAAAAATTGGTATCAGAGCCATGAATAGGTCTATGACCAAAA
CTCAAGAGGATAAAACCTCACCAAAATACGAAAGAGTTCTTAACTCTAAAGATAAAAGATCTTTCAAGATCAAAACTAGTTCCCTCACACCGGTGAC
GGGGATCGCATGCGACTCTTGTCGTTTTTCTGCATCTGTCCATGGTAAGTTAAATTGTAGTTTTGTATCAAGTTCTGTATTTTATGAAAATGTGTAT
TCTATGGATTAAGTGCTATTTGTTCTGTAGTCAACTCTTTAGTTAATCCTGCATTACTTTTGATTTAAAATTCACTGGATTGTTTCGAGATGGTTTC
GGATTTTTCGAGCCAATTTGGAGTTACTGTTTATTTAATTTTTCTTTTACATCGTGTAGTGCTTTTTTTTCCTTTTTTTTTATCTTTTTTTTTTTTT
TTTTTTTTTTTCTTTTGTCAAGGTGGAATAGGTATGGATTGGAAGTCGATAACGGATTGTTGTGCATTTGCGTGATGAATTTCCACCAACTTATCA
AAGGACTTATGTTCTTGAACGTATTTCTGAAAATACTGGTACTCTGCTTGGGCGGCTTTTAAATGCATCTGCAACTGATCTTGACCACTCGACTTTT
CCGTCCCTGGAATTCCGGAGAAGCTGTGGGGTTGAGTGGGGGCTAGAATTGGGTCGGTGAGGAACTGAGAAGTTTACTCCTTGACCATCGAAAGTGT
TCGTCCTCTTGTTTCGTCCGGCACTTGTCACCGTCGTTCGCGGCGACGAGGGAGTGCCAAAACCCAAATTCCGAGGGACACCGGTCGGCGCAGTTTT
GCGGTGTGAATATTACACCCCTACTTGTTGAAGTTGTACTCGCGTTTGAAATTTGTTGTTACAATTTTGTCGAAGCTTGTAAGAACCGCGAGCTCGT
AACATGACATAATTTTGTGAGAGAATGCTGGGTTTTGTATAGAAGTTGGTGGAAAAAGTTGATTGTAGATGGTCTCCACCGTCTTGGACCAACATTT
ATTACGAAAGTGAGAGGGAGGAAGAGAGAGAGAGAGCGAGAGAGAGAGCGGGAGAGAGAGATCGGCAATTCTTAAACCTTTTTCCGATCATTCGTTG
TCTGGAGCAATCAACAGAGAGAGCTCAAGGAGCGTGAGGAATGCGGGTTCGACCTGAAATAAGTTCGTTATTTTCGTTTAGGTCGGCTTAGCAGATG
AAGTCCGCTGAATTGCTGCTTGGACTGTCAATTGGTTGCTCCAGAGGCTCGATGGGGAGGAAGGTGTTTGGATGCGAATGAAGTCGTGTAAGGAGGT
TGAACGACTTTTGAGTGGCAAATTCGGTCATATGCTGGCAAGGATGGCTGCTTGGAGGAGTTTGGTTCCAGTGTCTTGGAGATTGTGTTGCAGGGAA
GTCTCTTCGGTAAGCAAATCATTAGTTTGTGGTTTGATCCAAATCGGGCAACATGTTGCCCTATCAAACTCGCTTGTCAAGGCACAGCGAAGATGTT
TTTACTCTTTTTAGGGTCACCATTCGTTGCTGGCTCATCACACACAATGCCAAGAGTTGTATTGTAATCGCGACTTAATCCATACACGTCTGCAGAC
ACAGAAAATTGGTCACGAAAATGGCCAGTATTTTGCTACCAGGCGATGGTATTCTCAACCATGGACGGCTCCCTACGATGCGGGGCAGCCTACGTCT
GAATCGCACCCTCAGGTATGCCTCTTTGCGTGGGTCTGAATGATGGAAGCGGAGAATCTGTTCTGGCATTTTGGAAGTTTCAGGTAACGAATTTGTG
CAATGCTGGAAAGTGATGTTAATCTGCATAAAGGTGGCAGATGCTGTCAGGGGATGAGCTGTTTCCCGGCATTAGTCTTCGAGATTTCAAGGAAAGA
ANAACAGCACTTCGNCNNCNT

G418 cassette sequence
3′flanking (targeting)sequence
Endogenous (genomic, non-targeted) sequence

New Phytologist

physcodillo1A/1B/2-line 16

>5′ insertion PCR product sequence

CGNGTTTCGCAGATAGTTTCCAGGTTCCGGAGATTCCAATACGGGAAATTTgTCaccCTGATTCTTGAGCTTGAGAGCTGGTGTGATGTGTTCgGGA
GAGAGTAGAGACGAGAGTTTGAACGATCACGACGACTGGCTCCGGTCGGAACGAGTGTTTGACAGTGGAAGGCTTGATCCAAATGTCTTGTGGCCTG
AGTGATGTTTTCGTGGTTGAGGGCTTTTCAACTCCGCAGAGACAGTTGGTGCTGAAAAGTCTGGGGAAGCAAGTGGGTAGATTCGAATTTGTGCCGT
TAGGTCTCCGTCGACACTGGAATTAGCGGTTACACGCCGAGTGAACTTGTTAGTGAGCATTCGGACAGACCATGCCCTAGCAATCTCGCTCGGTTAG
GCTCGTCATTTGGAAACGCTGAATCCGTCCGCAAAGCTTTTTCTCTTTCTCCCACTACCCCTACTCGTAATTCTCTCCCCACACACCTTCATTTGGG
CAGTATGGATGCCAATTTTCTTCCCGTTGACTACTTCAAATTTCCGTACTCAAAGTGTCCTCCTGACCTGTGACGCGTTGCCGAAGATATTGCTGGC
TGAATTCGGCGTCTCTCTGCAACGTAGGACAAATGCTTCAGAGTGCCCGGTCAAAGTAATACGCAGATGGATATCCCGTTTCTTCAACACGAAATTT
CAAACTAGCCACACGATAGTTCAAAACGGAGAAGTGAGAGGAGAAAACGACACCACAACGGCTCATCTCGAAGTTTGCGAAGATCCGAAACAAAGAA
GTTGGGGCATAGATATGAGGGGGCGCAGGATAGTGGATTCAAAGTGATATCTGGATTGGAGCGATCAACTAGAGGCAACAGTCAGATTGATCATGGG
ACAAGAAACATGAGGTCATTATAAAACCAGTACCACTGAAAAATATTCAACGATTTTGCTTCTCAACTTCTGGAACAAGTTTGGAAAGTATGTAGTA
TAATCAATTAATGGAGGGACAGCACATGAAATATCCAACAAGCGGCGGCGCGAAGTCAATGTGGCGGTGGACGATCAGGAGCAGCAGCAGACTGTT
GTCTATAAGAAAGCCAGGATAACATCGTCGCCGACGTCCGCTTGTGGTGCATCGGCGGTGCCCCCTATAGAATCAGGTGTTAAAATTGGCGCGGGTC
GCGATGCCCATTGGACAAGTCTGCCAGATGAAACCGTGCTTGGGTTGTTTAATCTGCTGAACCACAGGGACATGGCGAGCCTTGCGTCCGTGTGAAA
GGGATGGCAGGTGCTGGGGAGCTCGCAGTCGCTGTGGAATTCGTCGGATCTTCGGTTGCACACGTTGAATTCGGAGATGGTGTCTGCGCTGGCAGGA
CGATGCTCGAATTTGGAGGCGCTGAAGTTCCGCAGAGGTGCCTTTGCGAGCTCAATTGTGGGGTTGCAGGCGAAGGGGCTTAGGGAAATGAGCGGCG
ATTGCTGCAGCCAGCTGTCGGATGCAACCCTGTCCATGGTGGTCGCCCGGCATGCGAACCTGGAGAGCAAAGGCCTTTTGAACTACCGATCTCTCTC
CCCATCTCGATCTTCCTCCCTCTCGCTTTCGTGATTGATGTTAATCCCAGAGAATAAAGTCCACCTGACCCAATTTTGTAAAACAACTGCTTTGAAA
AGCTCCAGCATTCTCACACAGAATGAAGCATTGTGCGTGCTCGCGGTCCCAACAAGCACTGGCGAGTTTACGGCGAAAAAGTTCCTGGCGCAGGTAC
AACGTGAAAAAGTAGGAGAGTGCAATTCACTACAGAGAACTTTCCGAGCGAAGTCCATCAAATTTTGGGTTCTGGCACTGCCTCATCGCGACGAACG
ACGGTCCAACATGGTCAACGAAACCGAGAGAACAACTTTCTTGTTCGTCATCAGAGCTTCATTTCCGTTCTTACCAGACGCCGTTCTAGGGCGAAGT
CAACTCCAACAACAACAACAGCTTCCCTGAGATAAATGCCTTCTAAAAGAGAGTACTATTACCAGCTTGTGACAGCCGAGAACATTAAGAATTATAC
TACTTTAATTTCTCGAATTTATCAAAATAAGTCCAACAACATATGCATAAAAATAAATATAAATCATGCATATGCATAAAAATAAATCCGGATCCCC
CGGGCTGCAGGAATTCGATTCGAGGGGGGGCCCGGTACCCCTACTCCAAAAATGTCAAAGATACAGTCTCAGAAGACCAAAGGGCTATTGAGACTTT
TCAACAAAGGGTAATTTCGGGAAACCTCCTCGGATTCCATTGCCCAGCTATCTGTCACTTCATCGAAAGGACAGTAGAAAAGGAAGGTGGCTCCTAC
AAATGCCATCATTGCGATAAAGGAAAGGCTATCATTCAAGATGCCTCTGCCGACAGTGGTCCCAAAGATGGACCCCCACCCACGAGGAGCATCGTGG
AAAAAGAAGACGTTCCAACCACGTCTTCAAAGCAAGTGGATTGATGTGACATCTCCACTGACGTAAGGGATGACGCACAATCCCACTATCCTTCGCA
AGACCCTTCCTCTATATAAGGAAGTTCATTTCATTTGGAGAGGACAGCCCAAGCATGGAGAACCGACCTGCAGGCATGATTGAACAAGATGGATTGC
ACGCAGGTTCTCCGGCCGCTTGGGTGGAGAGGCTATTCGGCTATGACTGGGCACAACAGACAATCGGCTGCTCTGATGCCGCCGTGTTCCGGCTGTC
AGCGCAGGGGCGCCCGGTTCTTTTTGTCAAGACCGACCTGTCCGGTGCCCTGAATGAACTCCAAGACGAGGCAGCGCGGCTATCGTGGCTGGCCACG
ACGGGCGTTCCTTGCGCAGCTGTGCTCGACGTTGTCACTGAAGCGGGAAGGGACTGGCTGCTATTGGGCGAAGTgccGGGGCAGGATCTCCTGTCAT
CTCACCTTGCTCCTGCCGAGAAAGtaCTTNNNAANTTTCT

Endogenous (genomic, non-targeted) sequence
5′flanking (targeting) sequence
G418 cassette sequence

***physcodillo1A/1B/2*-line 16**

>3′ insertion PCR product sequence

```
TCTACAAATCTATCTCTCTCTATTTTCTCCCNNTTTAANGNGTGAGTAGTTTCCCGATAAGGGAAATTAGGGTTCTTATAGGGTTTCGCTCATGTGT
TGAGCATATAAGAAACCCTTAGTATGTATTTGTATTTGTAAAATACTTCTATCAATAAAATTTCTAATTCCTAAAACCAAAATCCAGTACTAAAATC
CAGATCTCCTAAAGTCCCTATAGATCTTTGTCGTGAATATAAACCAGACACGAGACGACTAAACCTGGAGCCCAGACGCCGTTCGAAGCTAGAAGTA
CCGCTTAGGCAGGAGGCCGTTAGGGAAAAGATGCTAAGGCAGGGTTGGTTACGTTGACTCCCCCGTAGGTTTGGTTTAAATATGATGAAGTGGACGG
AAGGAAGGAGGAAGACAAGGAAGGATAAGGTTGCAGGCCCTGTGCAAGGTAAGAAGATGGAAATTTGATAGAGGTACGCTACTATACTTATACTATA
CGCTAAGGGAATGCTTGTATTTATACCCTATACCCCCTAATAACCCCTTATCAATTTAAGAAATAATCCGCATAAGCCCCCGCTTAAAAATTGGTAT
CAGAGCCATGAATAGGTCTATGACCAAAACTCAAGAGGATAAAACCTCACCAAAATACGAAAGAGTTCTTAACTCTAAAGATAAAAGATCTTTCAAG
ATCAAAACTAGTTCCCTCACACCGGTGACGGGGATCGCATGCGACTCTTGTCGTTTTTCTGCATCTGTCCATGGTAAGTTAAATTGTAGTTTTGTAT
CAAGTTCTGTATTTTATGAAAATGTGTATTCTATGGATTAAGTGCTATTTGTTCTGTAGTCAACTCTTTAGTTAATCCTGCATTACTTTTGATTTAA
AATTCACTGGATTGTTTCGAGATGGTTTCGGATTTTTCGAGCCAATTTGGAGTTACTGTTTATTTAATTTTTCTTTTACATCGTGTAGTGCTTTTTT
TTCCTTTTTTTTTATCTTTTTTTTTTTTTTTTTTTTTTTTTCTTTTGTCAAGGTGGAATAGGTATGGATTGGAAGTCGATAACGGATTGTTGTGCAT
TTGCGTGATGAATTTCCACCAACTTATCAAAGGACTTATGTTCTTGAACGTATTTCTGAAAATACTGGTACTCTGCTTGGGCGGCTTTTAAATGCAT
CTGCAACTGATCTTGACCACTCGACTTTTCCGTCCCTGGAATTCCGGAGAAGCTGTGGGGTTGAGTGGGGGCTAGAATTGGGTCGGTGAGGAACTGA
GAAGTTTACTCCTTGACCATCGAAAGTGTTCGTCCTCTTGTTTCGTCCGGCACTTGTCACCGTCGTTCGCGGCGACGAGGGAGTGCCAAAACCCAAA
TTCCGAGGGACACCGGTCGGCGCAGTTTTGCGGTGTGAATATTACACCCCTACTTGTTGAAGTTGTACTCGCGTTTGAAATTTGTTGTTACAATTTT
GTCGAAGCTTGTAAGAACCGCGAGCTCGTAACATGACATAATTTTGTGAGAGAATGCTGGGTTTTGTATAGAAGTTGGTGGAAAAAGTTGATTGTAG
ATGGTCTCCACCGTCTTGGACCAACATTTATTACGAAAGTGAGAGGGAGGAAGAGAGAGAGAGAGCGAGAGAGAGAGCGGGAGAGAGAGATCGGCAA
TTCTTAAACCTTTTTCCGATCATTCGTTGTCTGGAGCAATCAACAGAGAGAGCTCAAGGAGCGTGAGGAATGCGGGTTCGACCTGAAATAAGTTCGT
TATTTTCGTTTAGGTCGGCTTAGCAGATGAAGTCCGCTGAATTGCTGCTTGGACTGTCAATTGGTTGCTCCAGAGGCTCGATGGGGAGGAAGGTGTT
TGGATGCGANTGANGTCGTGTAAGGAGGTTGAACGACTTTTGAGTGGCAAATTCGGTCATATGCTGGCAAGGATGGCTGCTTGGAGGAGTTTGGTTC
CAGTGTCTTGGAGATTGTGTTGCAGGGAAGTCTCTTCGGTAAGCAAATCATTAGTTTGTGGTTTGATCCAAATCGGGCAACATGTTGCCCTATCAAA
CTCGCTTGTCAAGGCACAGCGAAGATGTTTTTACTCTTTTTAGGGTCACCATTCGTTGCTGGCTCATCACACACAATGCCAAGAGTTGTATTGTAAT
CGCGACTTAATCCATACACGTCTGCAGACACAGAAAATTGGTCACGAAAATGGCCAGTATTTTGCTACCANGCGATGGTATTCTCAACCATGGACGG
CTCCCTACGATGCGGGGCAGCCTACGTCTGAATCGCACCCTCAgGTATGCCTCTTTGCgTGggTCTGAATGATGGAAGCggAcAATCTGcTCTGGCA
TTTtGgANGTTCCANGTAACNAATTTGNNCANTGCTGAG
```

G418 cassette sequence

3′flanking sequence

Endogenous sequence

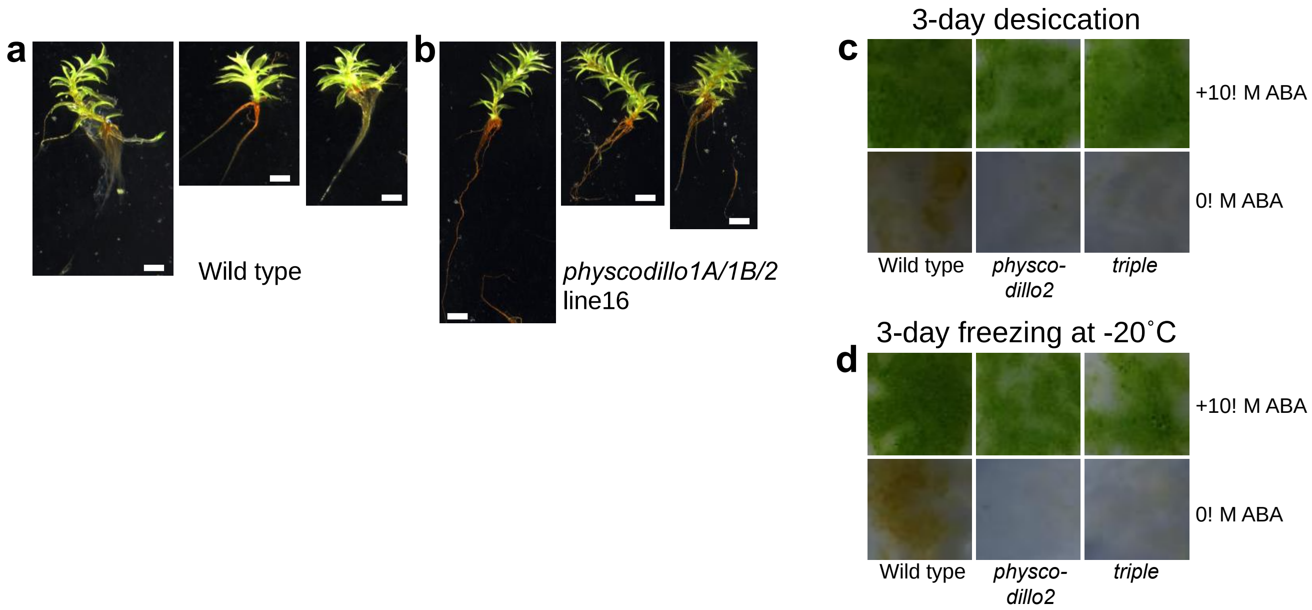

Fig. S4 *physcodillo* triple mutants show no obvious differences in rhizoid development or vegetative ABA responses. (a) Wild type plants. Bars, 5 mm. (b) *physcodillo* triple mutant line 16 plants. Bars, 5 mm. (c) Seven-day-old protonemal tissue was pretreated ± 10 µm ABA for 24 h, desiccated in a flow hood for 3 days, and then rehydrated for 3 days before assessing growth. No differences are seen between wild type, *physcodillo2* or *physcodillo* triple mutants in the presence or absence of ABA. (d) Seven-day-old protonemal tissue was pretreated ± 10 µm ABA for 24 h, frozen at -20°C for 3 days, and then placed at room temperature for 3 days before assessing growth. No differences are seen between wild type, *physcodillo2* or *physcodillo* triple mutants in the presence or absence of ABA. Experiments (c) and (d) were performed in triplicate: three biological repeats of each. Similar results were seen with 100 µM ABA.

Table S1 Primers used to generate and characterize *PHYSCODILLO1A/1B* gene replacement and generate *arabidillo* mutant rescue lines

**Amplification of 5' flanking sequence for gene replacement cassette**	
P1+3KO_5'F-2.XbaI	AAATCTAGAACTGGCTCCGGTCGGAACGAG
P1+3KO_3'R-2.BamHI	AAAGGATCCGTTCTCGGCTGTCACAAGCTGG
**Amplification of 3' flanking sequence for gene replacement cassette**	
P1+3KO_3'F.SphI	AAAGCATGCGACTCTTGTCGTTTTTCTGCATC
P1+3KO_3'R.EcoRV	AAAGATATCCCTGAAACTTCCAAAATGCCAG
**Primers to confirm 5' integration**	
P1+3KO5'F	AGTTTCCAGGTTCCGGAGAT
G418.R.319	TACTTTCTCGGCAGGAGCAAG
**Primers to confirm 3' integration**	
P1+3KO3'R	GGTAAACGAGACCGAAGTG
G418.F.341	CCATCATGGCTGATGCAATGC
**5' integration PCR product sequencing primers**	
35S_Reverse	TGATGGCATTTGTAGGAGCC
5'flank_seqF2	TTGCCGAAGATATTGCTGGC
5'flank_seqF3	AAATATCCAACAAGCGGCGG
5'flank_seqF4	CCCATCTCGATCTTCCTCCC
**3' integration PCR product sequencing primers**	
G418_F	TCGCCTTCTTGACGAGTTCT
pMBL10.vectorF	AAGTGGACGGAAGGAAGGAG
3'flank_seqF2	TGGGCGGCTTTTAAATGCAT
3'flank_seqF3	TCGTTTAGGTCGGCTTAGCA
**RT-PCR primers**	
P1-RT.GSP.F	GGCGCAATCGAAGCACTGGTGG
P1-RT.GSP.R	TGTACGTCCTCAAAATCAGAGTGC
P2-RT.GSP.F	CGCAATTGAAGCACTGGTGGATCT
P2-RT.GSP.R	ACGTCCTCAGAGTTCGAGTGTGC
PptubF	TGTGCTGTTGGACAATGAG
PptubR	ACATCAGATCGAACTTGTG
**Rescue line primers**	
P1/2-5'Start_BamHI	AAAGGATCCATGTCCAACAAGCGGCGGCG
P1_3'end_no_STOP_NotI	AAAGCGGCCGCCGACAGTGCCACCACCGTGTTGC
Sel.5'St.XbaI	AAATCTAGAATGCGTCGCGTTCGCCGGAAATGCG
Sel-St.NotI	AAGCGGCCGCGCTTTTCTCGCTCTGGCACAGCTCCA

Methods S1 Generation of PHYSCODILLO-GFP transgenic plants, construction of *arabidillo* mutant rescue lines, construction of *physcodillo1a/1b/2* triple deletion mutants and screening procedure.

Generation of PHYSCODILLO-GFP transgenic plants

The *PHYSCODILLO1* CDS was inserted in-frame with a GFP reporter gene in BamHI/HindIII cut *pHSP*::MCS-GFP (GenBank accession KP893621) to create *pHSP*::PHYSCODILLO1-GFP. The *pHSP*::PHYSCODILLO1-GFP cassette was then subcloned into pBS108CH-35SNPTb (GenBank accession GQ463722; Saidi *et al*., 2009) using a half blunt strategy using the NotI and Asp718/SmaI sites. The *PHYSCODILLO2* CDS was inserted in-frame with a GFP reporter gene in XmaI/HindIII cut *pHSP*::MCS-GFP to create *pHSP*::PHYSCODILLO2-GFP The *pHSP*::PHYSCODILLO2-GFP cassette was subcloned into pBS108CH-35SNPTb using a half blunt strategy using the NotI and Asp718/SmaI sites. As a control, the *pHSP*::MCS-GFP was similarly subcloned into pBS108CH-35SNPTb to create *pHSP*::MCS-GFP-10835SNPTb.

For stable expression, *pHSP*::MCS-GFP-10835SNPTb, *pHSP*::PHYSCODILLO1-GFP and *pHSP*::PHYSCODILLO2-GFP were transformed into wild type protoplasts and successful integration confirmed following two rounds of G418 selection.

Construction of *arabidillo* mutant rescue lines

35S::PHYSCODILLO1-GFP and 35S::SELAGIDILLO-GFP fusions were made in pGreen0029 by replacing ARABIDILLO1 in the 35S::ARABIDILLO1-GFP construct (Coates *et al*., 2006). *PHYSCODILLO1* and *SELAGIDILLO* cDNA sequences were amplified using Long PCR enzyme mix (Thermo Scientific; Waltham, MA, USA). The *PHYSCODILLO1* CDS was amplified using P1/2-5'Start_BamHI and P1_3'end_no_STOP_NotI and ligated into BamHI/NotI cut pGreen (containing 35S-GFP-ter; Coates *et al*., 2006) to create 35S-*PHYSCODILLO1*-GFP-ter. The *SELAGIDILLO* CDS was amplified using Sel.5'St.XbaI and Sel-St.NotI and ligated into XbaI/NotI cut pGreen (containing 35S-GFP-ter) to create 35S-SELAGIDILLO-GFP-ter. Primer sequences are outlined in Table S1.

Construction of *physcodillo1a/1b/2* triple deletion mutants and screening procedure

The *PHYSCODILLO1A/1B* double deletion construct was generated by cloning 5' and 3' homologous flanking sequences from *Physcomitrella* genomic DNA and inserting them into the pMBL10a vector either side of a G418 resistance cassette. The 5' flanking sequence was amplified using P1+3KO_5'F-2.XbaI and P1+3KO_3'R-2.BamHI and ligated into XbaI/BamHI cut pMBL10a to create pP1A+1BdblKO-5'. The 3' flanking sequence was amplified using P1+3KO_3'F.SphI and P1+3KO_3'R.EcoRV and ligated into SphI/EcoRV cut pP1A+1BdblKO-5'. The resulting construct was transformed into protoplasts isolated from the *physcodillo2* deletion mutant (Moody *et al*., 2012). Two rounds of G418 selection were carried out to identify putative transformants. To verify the presence of a G418 resistance cassette within the *PHYSCODILLO1A/1B* locus and confirm the generation of *physcodillo1a/1b/2* triple deletion mutants, PCR was carried out using GoTaq DNA Polymerase (Promega; Madison, WI, USA). 5' integration was confirmed using the primers P1+3KO5'F and G418.R.319 and 3' integration was confirmed using the primers P1+3KO3'R and G418.F.341. The PCR reactions were repeated 3 times and on one occasion the PCR products were sequenced (Fig. S3). RT-PCR was carried out twice to confirm loss of *PHYSCODILLO1* mRNA expression. Primer sequences are outlined in Table S1.

References

Coates JC, Laplaze L, Haseloff J. 2006. Armadillo-related proteins promote lateral root development in Arabidopsis. *Proc Natl Acad Sci, USA* **103:** 1621–1626.

Moody LA, Saidi Y, Smiles EJ, Bradshaw SJ, Meddings M, Winn PJ, Coates JC. 2012. *ARABIDILLO* gene homologues in basal land plants: species-specific gene duplication and likely functional redundancy. *Planta* **236:** 1927–1941.

Saidi Y, Finka A, Muriset M, Bromberg Z, Weiss YG, Maathuis FJM, Goloubinoff P. 2009. The heat shock response in moss plants is regulated by specific calcium-permeable channels in the plasma membrane. *Plant Cell* **21:** 2829–2843.
